# Exploring neurodevelopmental concerns: insights from a public neuropediatric learning disabilities multiprofessional outpatient facility in Brazil

**DOI:** 10.3389/fpsyg.2025.1363536

**Published:** 2025-01-28

**Authors:** Janaína Aparecida de Oliveira Augusto, Thalita Francielli Lopes Ferreira, Rodrigo Genaro Arduini, Talita Meneses de Almeida Bastos, Nádia Santana Pereira Campanha, Rita de Cássia Coutinho Vieira Fornasari, Patrícia Regina Flaviano Stella, Adriana Nobre de Paula Simão, Joyce Nelly Leal de Moraes, Sylvia Maria Ciasca

**Affiliations:** Laboratory for Research in Learning Disabilities and Difficulties, and Attention Deficit Disorder (DISAPRE), Medical Sciences Faculty (FCM), Neurology Department, Universidade Estadual de Campinas (UNICAMP), Campinas, Brazil

**Keywords:** learning disabilities, teaching, dyslexia, ADHD, child, intellectual disability, neurodevelopment, public health infrastructure

## Abstract

**Introduction:**

Specialized public services for attending children and adolescents with complaints of scholar difficulties are scarce in Brazil. It is important to recognize this target population and understand its demands, so these facilities may be able to meet their needs and offer qualified and effective services. The main objective of this study was to characterize the profile of neuropediatric patients cared for in a reference outpatient facility in Brazil.

**Method:**

Data were extracted from children’s and adolescent’s records, corresponding to assessments carried out between March 2017 to March 2023.

**Results:**

From 220 selected records, 70% had complaints related to learning difficulties and 79.1% to attention disorders. The most frequent diagnoses were attention-deficit/hyperactivity disorder (30%), intellectual deficiency (19.1%), and learning-specific disorder (17.7%). A significant association with scholarship was found between motor agitation [χ^2^(9) = 29.8; *p* < 0.001], behavioral complaints [χ^2^(9) = 16.2; *p* = 0.050], and language difficulties [χ^2^(9) = 17.0; *p* = 0.043]. Results have indicated significant differences relating to diagnosis and gender: boys had a higher prevalence for dyslexia [χ^2^(1) = 4.44; *p* = 0.035], intellectual deficiency [χ^2^(1) = 8.38; *p* = 0.004], and autism spectrum disorder [χ^2^(1) = 9.29; *p* = 0.002], when compared to girls.

**Conclusion:**

These results corroborate international findings over complaints regarding learning difficulties and correlated disturbances, in addition to the existing comorbidities between different diagnoses related to neurodevelopment and scholar acquisitions.

## 1 Introduction

In the Brazilian public healthcare system (Sistema Único de Saúde – SUS), university-linked hospitals are the main subjects of the development of specialized medical area structured programs, allowing specialties such as pediatric neurology, psychiatry, and neuropsychology to work together and, thus, allow a better integration among subspecialties (Gomes and Pedrero, 2015). As constitutionally declared (Brazil, 1988), healthcare is a governmental duty for all citizens. SUS was built under a hierarchized, tax-funded system, with universal access; i.e., every person residing permanently or temporarily within the Brazilian territory has the right to access it. Primary care is based on health promotion and basic attending, while secondary care allows access to medical specialties and rehabilitation programs, and simple hospitalizations for clinical and surgical uncomplicated treatments; whereas tertiary care is mainly based on hospital (mostly university-linked) and complex specialized care, including organ transplantations, for example (Brazil, 1990).

Studies that characterized public teaching psychological care clinics, from primary and secondary levels, refer that learning difficulties and/or behavioral problems are the main complaints of children and adolescents referred to these institutions, whether from schools, family demand, or other healthcare facilities (Borges et al., 2019; Neto et al., 2015).

School complaints present throughout academic development are related to two categories: school difficulties and learning disorders. The first refers to extrinsic factors, where failures in understanding the proposed content are due to pedagogical problems, adverse socioeconomic conditions, low parental education, and an unfavorable family environment (Moojen et al., 2016) or conditions specific to the child, which indirectly interfere with learning, such as psycho-emotional problems (Aro et al., 2021) (such as anxiety and/or depression disorders), chronic illnesses, presence of special needs (in motor or sensory spheres) (Al-Mahrezi et al., 2016) and others. Learning disorders are the result of intrinsic factors, caused by dysfunctions of the central nervous system, which affect the way the brain processes information and can cause persistent and significant difficulties in acquiring academic skills, even when instruction is adequate, i.e., are neurobiological in nature (Moojen et al., 2016; Lima and Ciasca, 2015).

Sei et al. (2019) have found a predominance of problems in the scholar environment (33.75%) in their sample of children and adolescents. The same results were found in later research, which analyzed 3.138 screening records, and scholar complaints were the fourth most frequent demand, especially among male gender (70.3%) (De Almeida Cavalcante and De Sousa Braz Aquino, 2018). Sehnem and Abati (2016) described the same findings regarding referrals to psychological care facilities.

For a better comprehension of this phenomenon, internal and external factors that could impact the development of children must be evaluated, such as a family history of learning difficulties (Erbeli et al., 2018), the subject’s gender (Hulme and Snowling, 2016; Chordia et al., 2019), prenatal and birth conditions (prematurity, extremely low birth weight, substance abuse during pregnancy, etc.) (Fill et al., 2018), and environmental conditions leading to social vulnerabilities which, added to intrinsic factors (Becker et al., 2017) can raise the probability of neurodevelopmental disorders prevalence.

Early identification and intervention for neurodevelopmental, psychiatric, and behavioral disorders were described by Correll et al. (2018) as a healthcare cost reduction strategy, besides improving individual quality of life. Furthermore, compared with neurotypical children, mental health conditions are related to recurrent and greater use of the healthcare system (Alaie et al., 2019; Reid et al., 2019).

Studies that explore the profile of complaints reported by parents of children and adolescents treated in public health services are scarce, however, understanding these data is essential to providing better care for the needs and demands of pediatric patients and their families, allowing healthcare professionals to know the challenges faced by this population; in addition, by identifying patterns and trends in the presented complaints, these professionals can adapt and improve the offered services, ensuring a more effective and patient-centered approach. Finally, characterizing the profile of complaints reported by parents is essential for the development of public policies and targeted interventions, thus promoting the well-being and integral health of children. Children and adolescents, especially in low- and middle-income countries.

The objective of this study was to characterize the complaints of relatives, healthcare professionals, and schools that referred children and adolescents to a neuropediatric multiprofessional outpatient facility in the Brazilian public healthcare system, through clinical records. Secondarily, an association between the most frequent complaints and data such as scholarship, final diagnosis, and gender was analyzed.

## 2 Materials and methods

This research was performed as an exploratory, retrospective, correlational, and descriptive study, with a non-probabilistic sample. Two hundred and forty physical reports, related to psychological, psycho-pedagogical, pediatric, and phono-audiological evaluations in the Laboratory for Research in Learning Disabilities and Difficulties, and Attention Deficit Disorder (DISAPRE), located in the Hospital de Clínicas da Universidade Estadual de Campinas, from March, 2017 to March, 2023 were analyzed. Variables considered were: child/adolescent gender, age, scholarship, public or private school attendance, history of grade repetition, and clinical data: main complaints, previous hospitalizations, daily use of medications, previous multi-professional evaluations, and final diagnosis. Family history of scholarly difficulties and/or neurodevelopmental disorders, educational degree, and age of the informants were also collected.

**Figure 1:**
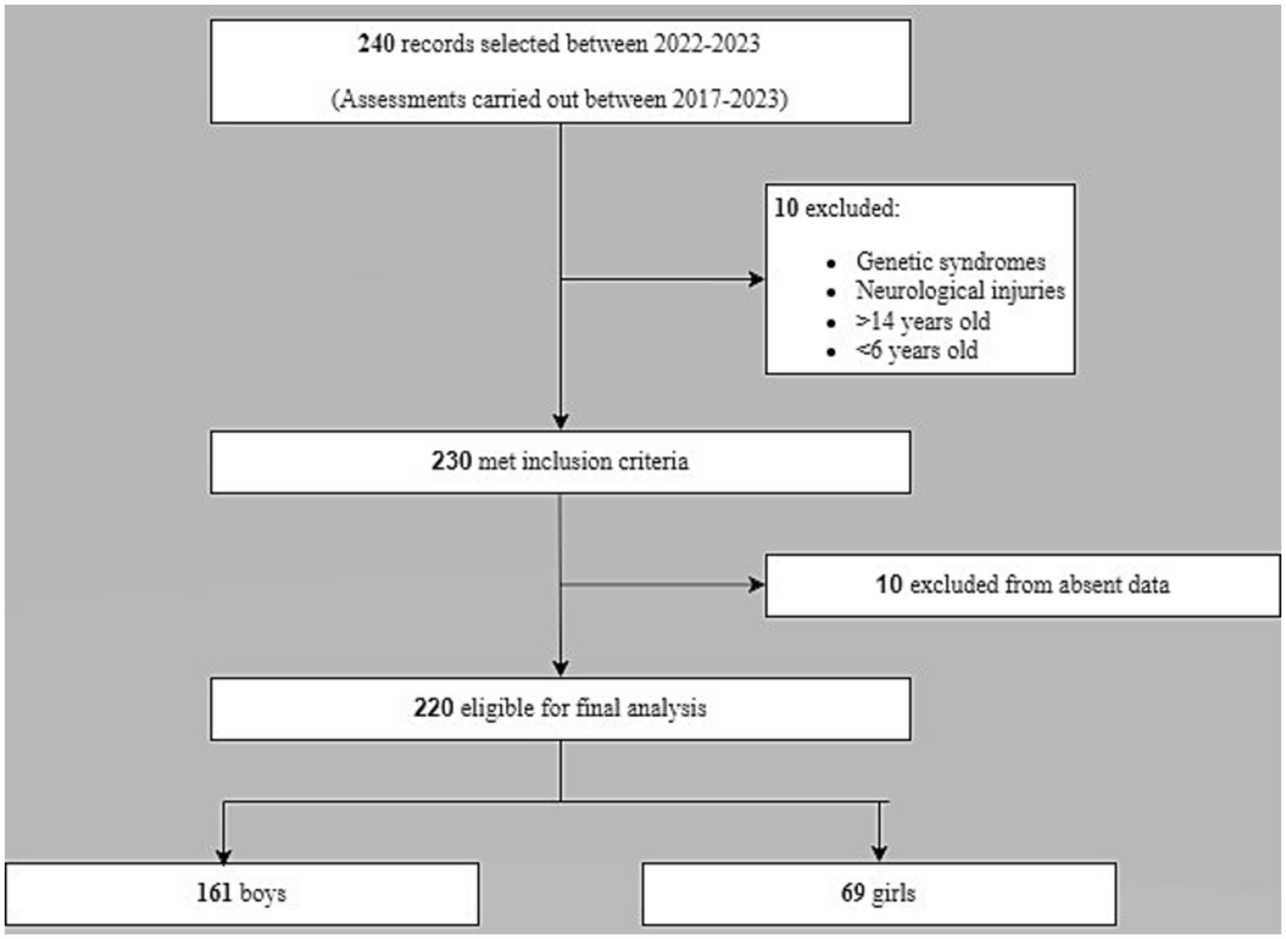
Data selection chart flow.

Inclusion criteria for data selections were: referral to the outpatient facility among the selected period, and ages between 6 to 14 years by the time of evaluation. Exclusion criteria: incorrect data filling in the records, patients with genetic diseases or syndromes, and a history of neurological injuries. In the final analysis, 220 records were selected (Figure 1).

This study was approved by the Ethics Committee, under #4.632.789. All ethical principles for medical research involving human subjects according to the Declaration of Helsinki were followed.

### 2.1 Statistical analysis

Data were analyzed with SPSS version 25.0 software. The chi-square test was used to verify the association between scholarship and the referred complaints from caregivers, and between gender and final diagnosis. To better elucidate the results, Cramer’s V and Phi effect sizes of statistically significant results (*p* < 0.05) were reported, in addition to the odds ratio. Finally, descriptive analyses were used to characterize the sample, frequency of complaints, and clinical history of the selected data.

## 3 Results

### 3.1 Social and demographic data analysis

**Figure 2:**
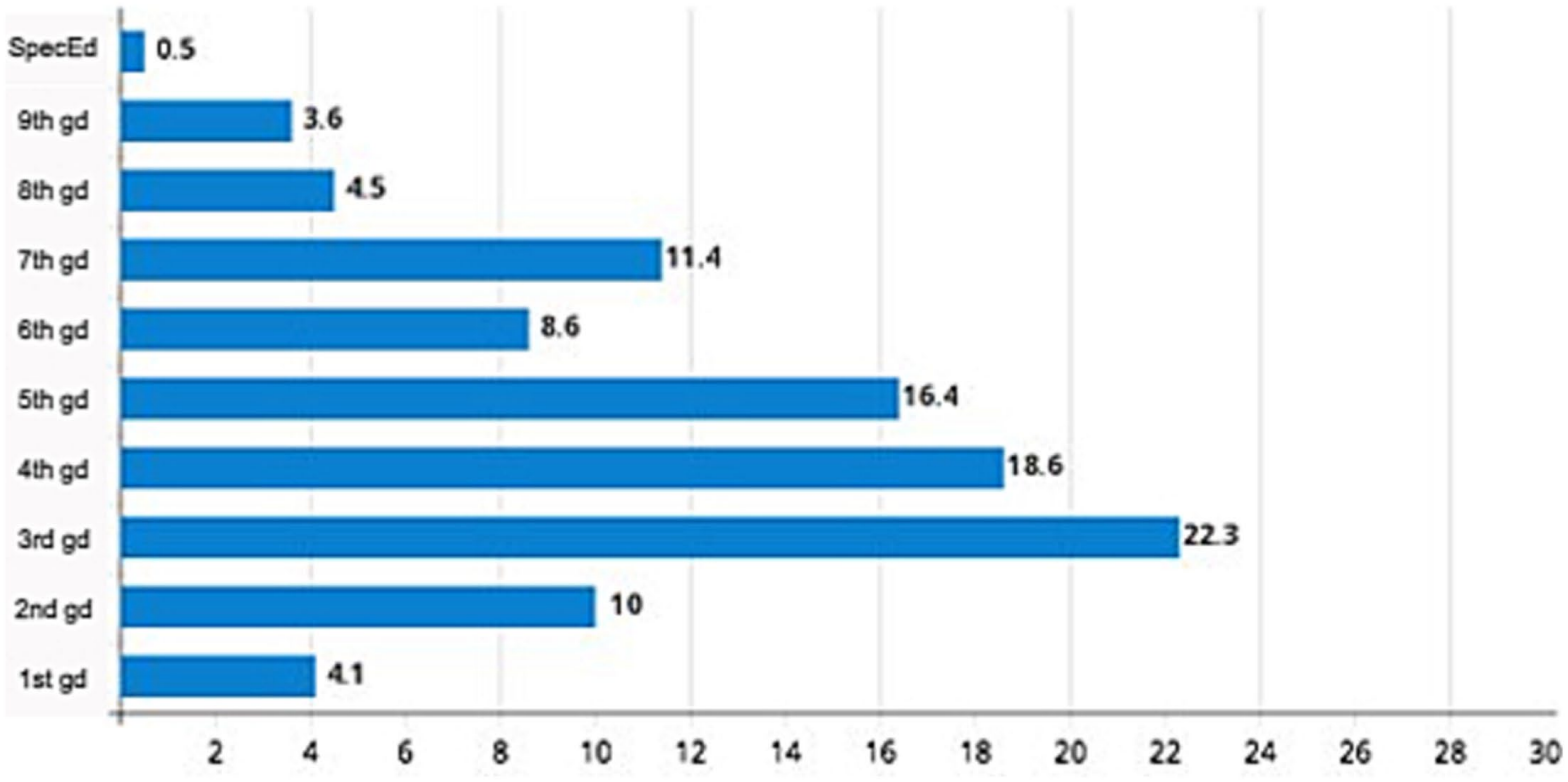
Scholar attendance of the subjects. SpecEd, special education; gd, grade.

As shown in Table 1, from the 220 selected records, 68.6% of the patients were male, with a mean age of 10.1 years (SD: 2.14); 22.3% were enrolled in 3rd grade (Figure 2), 86.8% attended public schools, and 32.3% had a history of grade repetition. Besides, 58.5% of the sample was evaluated from March 2017 to December 2018, since the COVID-19 pandemic led to a significant reduction of attendance in the outpatient facility, due to sanitary restrictions and school closures in Brazil. Regarding the informants’ data, 84.5% of the anamnesis were answered by the participants’ mothers, 8.7% by fathers, and 6.8% by other family members (grandparents, uncles, stepmothers, etc.), with a mean age of 40.2 years (SD: 8.07). Regarding education, 53.8% of the parents declared having completed High School, 29.1%, had Elementary School, and 17.1% had Undergraduate Degree.

**Table 1:** Social and demographic characterization of subjects.

Variables		% (*n*)
Gender	Female	31.4 (69)
	Male	68.6 (151)
School origin	Public	86.8 (191)
	Private	13.2 (29)
Scholar retention history	No	67.7 (149)
	Yes	32,3 (71)

### 3.2 Participants’ clinical history

Data analyzed from the selected records showed that 64.1% of all assessed children and adolescents did not have neurodevelopmental complaints regarding delay in acquisitions; however, speech delay was found in 17.7% of the subjects, associated motor and speech delay in 10.9%, and isolated motor delay in 7.3% of the sample.

According to caregivers’ reports, 70.9% of the subjects had already been submitted to some kind of intervention (psychological in 37.3%, psycho-pedagogical in 28.2%, phono audiological in 43.6%, neuro pediatric for 24.1%, psychiatric in 7.7% and occupational therapy for 6.4%). Physical therapy evaluation was reported only by 2.7%. Furthermore, 20.9% were already taking some kind of continuous medication, especially Risperidone and Ritalin.

During the evaluation period, 19 children had some kind of support at school, with 63.2% receiving tutoring, 31.6% being monitored by a specialized educational assistance service, and 5.2% being accompanied by an assistant teacher in the classroom.

Clinical history extracted from the reports also indicated that 22.3% had already undertaken central auditory processing analysis, 13.6%, had head computed tomography; 26.4% had an electroencephalogram, 7.7%, had a central nervous system magnetic resonance imaging, 15.5% had undergone ophthalmologic and 2.7% otorhinolaryngologic evaluations. In addition, 17.3% had neurological, 15.9%, had phono-audiological, 15% psychological, 8.2% psycho pedagogical, and 1.8%, had psychiatric evaluation.

Regarding family history, 43.2% of the caregivers pointed to some kind of substance abuse in their kinship; 19.1% had intellectual disorders, 14.5% had epilepsy, 14.2% had a depression background, 9.6% some sort of sensitive deficiency (auditory or visual), 7.7% for anxiety disorder, 8.6% for other psychiatric conditions (e.g., panic syndrome), 6.4% schizophrenia, 5.9% cerebral palsy, 5.5% autism spectrum disorder and 2.3% dyslexia.

### 3.3 Characterization of complaints and diagnostic hypothesis

After assessment in our outpatient facility, children and adolescents are referred to neurologists, psychiatrists, psychologists, speech therapists, pedagogues, and schools. As shown in Table 2, 70% of all complaints were related to learning difficulties, especially reading and writing. Seventy-nine percent of the caregivers pointed inattention and concentration disorders, 46.4%, calculating difficulties, 40.9% declared the presence of behavioral issues (aggressivity, difficulty to follow rules, impulsivity), and 40.9% of the subjects seemed not able to memorize the content offered during classes.

**Table 2:** Associations between complaints and scholarship.

Complaints	*n*	%	Scholarship (χ^2^/*p*)
Reading and writing difficulties	154	70.0	14.5 (0.107)
Mathematical difficulties	102	46.4	7.91 (0.543)
Attention and concentration difficulties	174	79.1	9.14 (0.424)
Motor agitation	53	24.1	29.9 (<0.001)
Emotional issues	73	33.2	16.0 (0.066)
Social interactions difficulties	45	20.5	7.39 (0.597)
Behavioral complaints	90	40.9	16.2 (0.050)
Language difficulties	41	18.6	17.0 (0.043)
Memorizing difficulties	90	40.9	8.21 (0.513)
Motor skills difficulties	26	11.8	13.3 (0.151)
Text interpretation difficulties	79	35.9	10.9 (0.280)
Stereotipies	22	10	6.62 (0.676)

χ^2^, chi-square; *p*, significance level.

A significant association between motor agitation and scholarship was found [χ^2^(9) = 29.8; *p* < 0.001; Cramer’s V = 0.368], where children attending 2nd grade had a probability 1.38 times higher than those in 1st grade to show this behavioral complaint; just like 3rd graders had a chance 2.89 times higher of being perceived as agitated by their relatives than 5th graders.

Behavioral complaints and scholarship also had shown a statistically significant association [χ^2^(9) = 16.2;*p* = 0.050; Cramer’s V = 0.276]: 2nd-year students presented 1.8 times more behavioral complaints when compared to those attending 1st grade; adolescents in the 7th grade were 1.4 times more pointed as agitated than their closely related 6th or 8th graders.

Oral language difficulties and scholarship [χ^2^(9) = 17.0;*p* = 0.043; Cramer’s V = 0.278] were also a significant correlation in our series. Second-year students were 0.69 times more likely to have complaints related to speech disorders than 1st-year attendees; 3rd graders had a 0.68 times greater chance than 4th graders for the same complaint. For all associations found, the magnitude of the effect was large.

**Figure 3:**
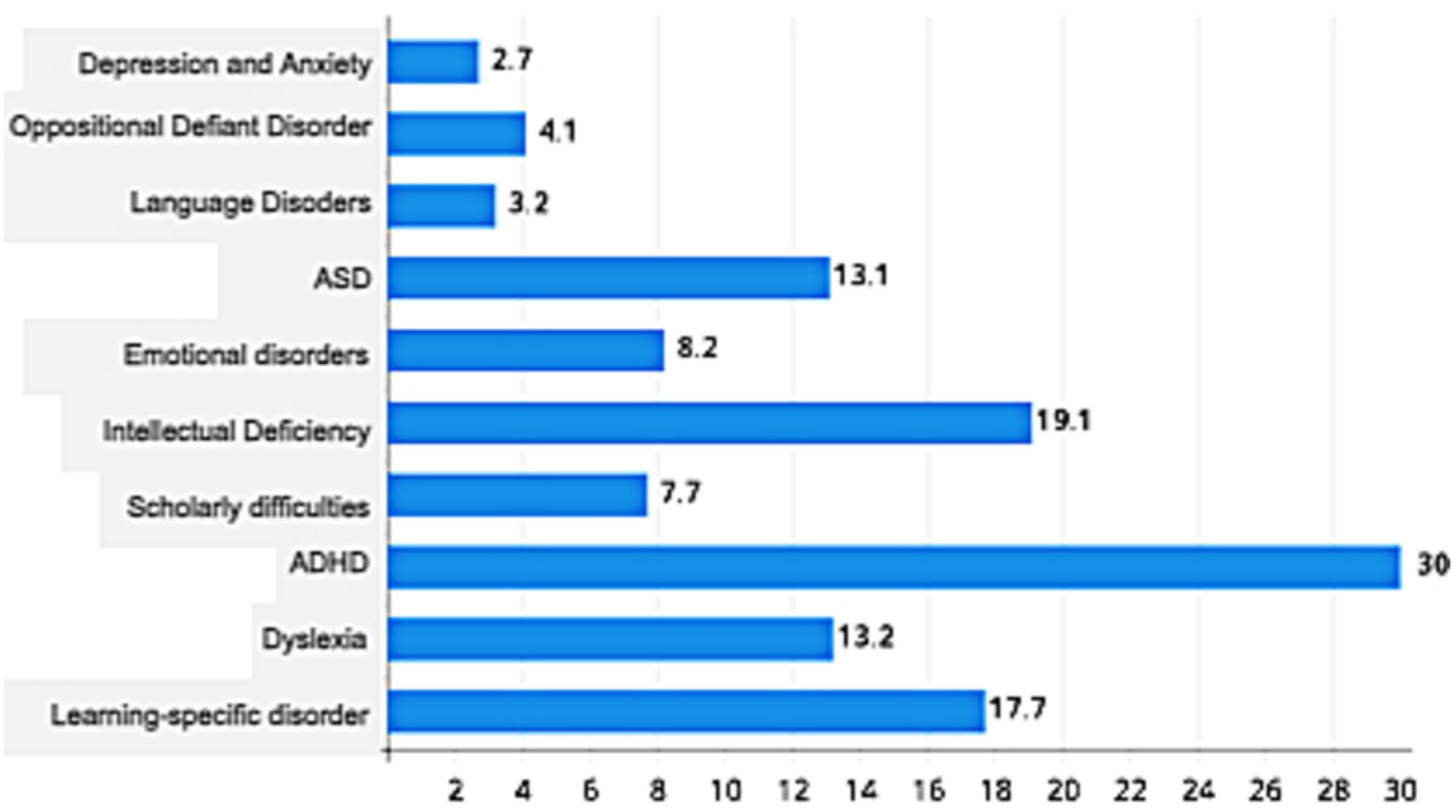
Diagnostic hypotheses frequency. ASD, autism spectrum disorder; ADHD, attention-deficit/hyperactivity disorder.

The most common diagnostic hypotheses raised by the multi-professional team were: attention-deficit/hyperactivity disorder (ADHD) – 30%, intellectual deficiency (19.1%), and learning-specific disorder (17.7%), as shown in Figure 3.

Comparisons between male and female genders were made to evaluate any differences regarding the hypotheses sought (Table 3). Results indicated that boys had a 0.44 times higher chance of a dyslexia diagnosis than girls [χ^2^(1) = 4.44;*p* = 0.035; 𝝓 = 0.142]; as for intellectual deficiency [χ^2^(1) = 8.38;*p* = 0.004; 𝝓 = 0.195], and autism spectrum disorder (ASD) [χ^2^(1) = 9.29; *p* = 0.002; 𝝓 = 0.205]. For all associations found, the magnitude of the effect was small.

**Table 3:** Associations between diagnostic hypotheses and gender.

Diagnoses	χ^2^	*p*	OR*
Learning-specific disorder	0.220	0.639	1.15
Dyslexia	4.44	0.035	0.44
ADHD	0.733	0.392	1.31
Scholarly difficulties	0.525	0.469	1.50
Intellectual deficiency	8.38	0.004	0.36
Emotional issues	1.56	0.212	0.53
ASD	9.29	0.002	7.33
Communication disorders	0.444	0.505	0.50
Oppositional defiant disorder	1.79	0.181	5.00
Depression	0.657	0.418	0.33
Anxiety	0.922	0.337	-

χ^2^, chi-square; *p*, significance level; OR, Odds ratio; ADHD, attention-deficit/hyperactivity disorder; ASD, autism spectrum disorder. ^*^All results regarding male gender.

## 4 Discussion

### 4.1 Sample demographic profile

The results found in this analysis corroborate previous literature findings, which indicate a higher prevalence of male gender referral to mental health services (Machado et al., 2014; Duarte et al., 2019; Sei et al., 2019). Boys are prone to present more externalizing behavioral complaints, such as aggressivity and inattention, that may cause losses in the social and academic spheres (Machado et al., 2014; Lundin et al., 2020).

Concerning scholarship, most of the children evaluated in the outpatient facility were attending 3rd to 5th grades, which relates to the late phase of Elementary School in our country. This result may reflect, on one hand, the requirements for the alphabetization cycle which, according to Brazilian guidelines and foundations for teaching law (Brazil, 2019), establish that all students shall be able to read and write by the end of 3rd grade (ages 8–9 years old). This demand generates concern among teachers with some students who are not able to reach this goal within the designated schedule (Corso and Meggiato, 2019).

On the other hand, referral in the later years of Elementary School for evaluation shows that teachers, school staff, healthcare workers, and caregivers in Brazil are not properly clarified about the warning signals regarding learning difficulties and/or neurodevelopmental disorders, which reinforces the teaching maintenance, by waiting for the student to reach 8 years of age and, then, start the process of referral and/or intervening, giving little emphasis in prevention and early identification (De Almeida et al., 2016).

Furthermore, the delay in early intervention may cause negative effects throughout the academic life of an affected student, e.g., poor educational performance, lower levels of College Education, and higher levels of psychological suffering, amongst others (De Beer et al., 2014; Piccolo et al., 2017).

The high prevalence of mothers as main informants in the anamnesis interview reinforces and corroborates studies that point out the role of women as the key caregivers of children with neurodevelopmental disorders or suspected abnormalities (Oliveira and Poletto, 2015; Cerqueira et al., 2016).

The results obtained demonstrate the relationship between the different aspects of child neurodevelopment and schooling, especially when verifying that the difficulties encountered increase in some cases for children who have left the early years of formal education.

### 4.2 Clinical history of the subjects

As identified in this sample results, developmental delays are frequently observed in neurodevelopmental disorders, such as ASD and intellectual deficiency (Goodwin et al., 2018; Matos et al., 2022). Subjects with subtle delays or soft neurological alterations tend late diagnosis, which favors a delayed intervention and poor prognosis (Coelho-Medeiros et al., 2019).

Evidence shows that a positive family history, like the ones identified in this study, significantly raises the chance of occurrence of neurodevelopmental disorders (ASD, ADHD, intellectual deficiency). Such findings contribute to the evidence of a direct influence of hereditary aspects for these conditions (Jokiranta-Olkoniemi et al., 2016).

Studies that evaluated the family saga for reaching a diagnosis for neurodevelopmental disorders indicate that caregivers may be encouraged by healthcare professionals to initiate an early intervention even without a previous diagnosis (Augusto, 2021; Rivard et al., 2021), as also shown in this study. Referral before a final diagnosis may improve the gravity of symptoms and aid in the identification of comorbidities. However, Augusto (2021) verified that, despite most of the children evaluated in his sample having undergone previous interventions, they did not favor a faster diagnosis. In this sense, the presence of qualified and properly trained professionals is a key aspect in the evaluation, as far as in the intervention processes.

Alternatively, the support schools offer for students with academic difficulties, as the ones identified in this study, aims to reduce pedagogical factors that impact the learning process (Augusto, 2021), and may be seen as a way of trying to reduce the delay in acquiring writing and reading skills.

### 4.3 Complaints and diagnostic hypothesis characterization

Regarding the reported complaints, there is literature consensus that the assertions of children’s and adolescents’ caregivers are mostly related to learning difficulties, behavioral problems (aggressivity, attention), agitation, and emotional aspects (anxiety, self-esteem, irritability) (Sei et al., 2019).

Our results have shown a higher concentration of complaints related to agitation and behavioral and language problems for children attending the earlier years of Elementary School. Cunha and Benetti (2009) described that the initial years of education may contribute to the identification of such complaints due to the new demands and challenges that are required in this cycle, especially for alphabetization.

In consonance with previous studies, this research has also found a higher prevalence of ADHD and ASD diagnoses. About ADHD, earlier descriptions were able to identify that this condition, in childhood, is more often identified in mental healthcare services, which generates greater financial demands for healthcare services around the world, corroborating the results in this analysis.

Regarding the correlations found between gender and neurodevelopmental disorders, recent studies indicate that males are up to four times more likely to be diagnosed with these conditions than females (WHO, 2019; Associação Americana de Psiquiatria, 2022). Bölte et al. (2023) add to the discussion that related behavioral expressions inherent to neurodevelopmental conditions are modulated by sex and gender in different but clinically important ways. For example, Lundin et al. (2020) described that men are more prone to present with externalizing problems, as those related to oppositional defiant disorder, as long as women are more prone to present with internalizing problems, like anxiety and mood disorders.

Despite the lack of studies correlating learning-specific disorders and gender, Landerl and Moll (2009) was able to identify that girls had a better performance in writing and reading skills when compared to boys. Similar results were observed by Arnett et al. (2017). As for ASD, gender differences are a focus in several recent studies, and aspects such as social camouflage have been pointed out as a contributing factor for male preponderance regarding the diagnosis of this condition.

This study was able to determine and describe the profile of complaints and diagnostic hypotheses of a Brazilian multiprofessional reference outpatient facility. Results obtained indicated that most of the complaints pointed out by caregivers were related to scholarly demands (reading, text interpretation, writing, and mathematical skills), behavior (attention, aggressivity, agitation), and also emotional aspects. These were the same complaints described in earlier studies, as a common demand in mental health facilities that care for children and adolescents.

Identifying the main complaints reported by parents is fundamental to planning interventions and using effective strategies that can contribute to the learning development of children and adolescents. In this sense, the use of digital interventions, such as the GraphoGame application in Portuguese, sponsored and adopted by Brazil’s Ministry of Education (MEC) and developed by a joint of Brazilian and Finnish researchers, to assist the literacy development of children in preschool and in the first years of alphabetization, is an evidence-based method which may promote the development of reading skills and writing, contributing to improvements in the learning process (GraphoGame, 2024). Another excellent tool is Comprehension Game (CGWorld Learning Ltd, 2024), an online platform that offers games and interactive activities to improve reading comprehension and cognitive skills for Finnish and English readers. It is aimed for educators and students, and it promotes learning in a playful way, with scientific basis. Its main objective is to allow readers to fully comprehend and retain what texts present, enhancing knowledge and promoting independence and citizenship.

Recently, aiming for better outcomes in the learning processes and social skills development, artificial intelligence tools (AIT) have been developed for children and adolescents with neurodevelopmental disorders, in an attempt of providing a more individualized approach for interventions in these populations. Although some educational practices are already well established for usage in scholar environments, especially for dyslexic students (Snowling et al., 2012), AIT will be able to focus on specific deficits these individuals present, like social skills demands for those with ASD (Barua et al., 2022).

Also, for reading proficiency and habit construction, the possibility of accessing online public libraries or free books and texts repositories (such as: Fundacao Educar.org, and Brazil’s MEC program “Conta pra mim”), (Fundação Educar, 2024; Brazil, 2018) is an important feature to be considered by public educational policy makers, mainly in low development countries, as the acquisition of low cost cell phones, connected online with public wi-fi zones, could bring for rural and/or vulnerable communities the possibility of discovering the joy of literacy.

The most prevalent diagnoses were: ADHD, intellectual deficiency, learning-specific disorder, and ASD. It is noteworthy that ADHD is considered, in the current literature, as the most frequent neurodevelopmental disorder diagnosed in childhood; in addition, leading to higher financial costs for mental health services. However, recently there has been a substantial increase in the diagnosis of other neurodevelopmental disorders, mainly ASD (Maenner et al., 2023).

In our referral, there were a few cases with a diagnosis of dyslexia, which is a separate entity when compared to the general group of complaints for specific learning disorders.

In Brazil, the described conditions above are still poorly understood by different healthcare and education professionals, who are the ones dealing directly with children and adolescents carrying the disorders. This certainly causes a delay when referring these subjects either to the public or to particular services.

It is necessary to increasingly disseminate survey results to obtain objective data that may solve problems involving children with any disorders and who require a careful and significant look for appropriate interventional referral.

By comparing the complaints described here with their own, global services may be able to exchange positive and negative experiences, saving time and resources in the path of improving care and welfare for neuroatypical children and their relatives and/or caregivers.

Despite the relevant results described in this research, some limitations are observed, such as the description of complaints based exclusively on reports from parents and caregivers, parental memory bias, the lack of detailed information about previous interventions and assessments to which the child was exposed (frequency, focus of the intervention), and a selection bias, as some participants had to be excluded due to incomplete data and/or if they were already diagnosed with genetic syndromes. Furthermore, retrospective data collection can be influenced by the quality of record keeping and subjective interpretation of clinical notes. For future research, a longitudinal follow-up study is suggested to verify the evolution of complaints reported after evaluation in health services.

## Resource identification initiative

SPSS (RRID:SCR_002865).

ChatGPT (RRID:SCR_023775).

Grammarly (RRID:SCR_023778). STATISCAL ANALYSIS’ section 2.1, for SPSS. Chat GPT and Grammarly’s RRID where added when mentioned, in the ‘METHODS’ section 2.

## Data Availability

The raw data supporting the conclusions of this article will be made available by the authors, without undue reservation.
